# Accuracy of *H. pylori* fecal antigen test using fecal immunochemical test (FIT)

**DOI:** 10.1007/s10120-021-01264-8

**Published:** 2021-11-18

**Authors:** S. A. V. Nieuwenburg, M. C. Mommersteeg, L. M. M. Wolters, A. J. van Vuuren, N. Erler, M. P. Peppelenbosch, G. M. Fuhler, M. J. Bruno, E. J. Kuipers, M. C. W. Spaander

**Affiliations:** 1 Departments of Gastroenterology and Hepatology, Erasmus MC University Medical Center’s, (Room Na-610) Gravendijkwal 230, 3015 CE Rotterdam, The Netherlands; 2 Department of Gastroenterology and Hepatology, Albert Schweitzer Hospital, Dordrecht, The Netherlands; 3 Department of Biostatistics, Erasmus MC University Medical Center, Rotterdam, The Netherlands

**Keywords:** *H. pylori*, Screening, Cancer, Fecal immunochemical test

## Abstract

**Background:**

Gastric and colorectal cancer (CRC) are both one of the most common cancers worldwide. In many countries fecal immunochemical tests (FIT)-based CRC screening has been implemented. We investigated if FIT can also be applied for detection of *H. pylori,* the main risk factor for gastric cancer.

**Methods:**

This prospective study included participants over 18 years of age referred for urea breath test (UBT). Patients were excluded if they had used antibiotics/bismuth in the past 4 weeks, or a proton pomp inhibitor (PPI) in the past 2 weeks. Participants underwent UBT, ELISA stool antigen test in standard feces tube (SAT), ELISA stool antigen test in FIT tube (Hp-FIT), and blood sampling, and completed a questionnaire on user friendliness. UBT results were used as reference.

**Results:**

A total of 182 patients were included (37.4% male, median age 52.4 years (IQR 22.4)). Of these, 60 (33.0%) tested *H. pylori* positive. SAT and Hp-FIT showed comparable overall accuracy 71.1% (95%CI 63.2–78.3) vs. 77.6% (95%CI 70.4–83.8), respectively (*p* = 0.97). Sensitivity of SAT was 91.8% (95%CI 80.4–97.7) versus 94.2% (95%CI 84.1–98.9) of Hp-FIT (*p* = 0.98). Serology scored low with an overall accuracy of 49.7% (95%CI 41.7–57.7). Hp-FIT showed the highest overall user convenience.

**Conclusions:**

FIT can be used with high accuracy and sensitivity for diagnosis of *H. pylori* and is rated as the most convenient test. Non-invasive Hp-FIT test is highly promising for combined upper and lower gastrointestinal (pre-) cancerous screening. Further research should investigate the clinical implications, benefits and cost-effectiveness of such an approach.

**Supplementary Information:**

The online version contains supplementary material available at 10.1007/s10120-021-01264-8.

## Introduction

*Helicobacter pylori* (*H. pylori*) is the most important risk factor for intestinal type gastric adenocarcinoma and classified as a class 1 carcinogen by the World Health Organization (WHO) [1]. Current practice recommends eradicating *H. pylori* when identified to prevent *H. pylori*-associated disease [2]. Some studies even advocate a “screen-and-treat” program to reduce gastric cancer burden [3]. However, in low incidence gastric cancer regions the low prevalence rates of *H. pylori* infections limit cost efficiency of such a strategy [4]. For high incidence regions, screening might be effective [5–7].

Several non-invasive diagnostic tests are already available. The urea breath test (UBT) has the highest sensitivity (90–96%) and specificity (88–98%) and has similar accuracy to the stool antigen tests (SAT) using ELISA (enzyme immune assay). Serology testing for *H. pylori* antibodies is easy to perform, but does not distinguish between active or prior infection, as antibodies can persist in the blood after eradication [2]. Previous studies compared invasive and non-invasive methods for the diagnosis of *H. pylori* in terms of sensitivity and specificity [8, 9]. Importantly, for a test to be effective, patient preference and acceptance are just as important as test performances [10].

Fecal immunochemical tests (FIT) are used in colorectal cancer (CRC) screening and are known for their ease of use [11]. Simultaneous non-invasive screening for gastric and colorectal cancer is potentially very attractive, in particular in populations with a higher incidence of both cancers. In addition, it may be cost-effective [12, 13]. FIT sampling may be a suitable medium for both goals, but also for clinical purposes to diagnose Hp infection by a non-invasive, easy to perform test at home. However, the potential to determine the presence of fecal *H. pylori* stool antigen in FIT has thus far not been investigated. While analyses of the fecal microbiome have already been proven promising and feasible in FIT and feces, suggesting that FIT might be a good tool to study bacterial presence [14, 15].

We therefore investigated whether *H. pylori* stool antigen can be detected in FIT and how this outcome relates to the other non-invasive *H. pylori* tests. Furthermore, we assessed patient preferences for these tests.

## Methods

### Study design

This prospective study was performed in two hospitals (one academic and one regional) in the Netherlands. Patients were eligible for inclusion if they were over 18 years of age and were referred for UBT at the general practitioner’s discretion. They were identified through the outpatient clinic of the participating hospitals and contacted by telephone. After informed consent, patients were sent a first questionnaire and feces sampling kits with instructions. Patients were excluded if they had used antibiotics/bismuth in the past 4 weeks, or a proton pump inhibitor (PPI) in the past 2 weeks. All participants underwent UBT, SAT, Hp-FIT and blood sampling. Patient inclusion took place between February 2018 and December 2020. UBT results were used as reference. The performers of the SAT, Hp-FIT and blood sampling were blinded to the UBT results. The institutional review boards of both participating hospitals approved the study (MEC-2017-528). This trial was registered in the Dutch trial register (NTR7052). All co-authors had access to the study data and had reviewed and approved the final manuscript.

### Baseline data collection

All participants completed two questionnaires, one before and one after performing the tests. The first questionnaire concerned details about age, sex, ethnicity and items about lifestyle factors, medical history, family history and medication use. Expected convenience and burden of the tests were assessed. The second questionnaire was handed out after all tests had been performed and included questions about actual experienced convenience and burden of each test.

Expected and actual experienced convenience and burden were asked in the following manner: patients were asked about pain, embarrassment and overall burden for all tests. All aspects had to be rated on a scale from zero to four: zero being “not at all painful/embarrassing /burdensome and four being extremely painful/embarrassing /burdensome (S1).

### Sampling collection

Feces sampling for Hp-FIT and SAT was performed at home on the same stool and collected within 24 h of the scheduled UBT. Patients were instructed to keep the stool at – 4 °C until the hospital visit. A blood sample was drawn during the hospital visit. Feces and blood samples were stored at – 80 °C until analysis. Serological testing of *H. pylori* antibodies was performed by commercial ELISA tests *(H. pylori IgG* ELISA, Gastropanel, Biohit Oyi, Helsinki Finland). All tests were performed according to the manufacturer’s instructions, which allowed for a one-time – 80 °C storage and defrosting.

### Serum samples

Serum samples were diluted 1:200 in sample diluent, 100 µl of this solution was added to the *H. pylori* antigen-coated microplates. After 30 min of incubation, samples were washed, and the conjugate solution was added. After another 30 min of incubation, the samples were washed and the substrate solution was added. Quantification of the optical density was performed using a spectrophotometer (Infinite M Nano Tecan group ldt.; Mannedorf, Switzerland) at a wavelength of 450 nm. For the serology test, a cutoff of 30 EIU was used as per the manufacturers’ protocol.

### Stool antigen ELISA

For the stool antigen ELISA, a commercial kit was used (Fecal *Helicobacter pylori* Antigen, ref KT 826, Epitope Diagnostics Inc.; San Diego, USA). In short, 40 mg of fecal material was suspended in 1 ml of assay buffer. A total of 100 µl of this sample was added to monoclonal antibody-coated microwell plates and incubated for 60 min. After washing the plate, the tracer antibody was added to the wells and incubated for 30 min. The plates were washed again and the HRP substrate was added for 10 min to develop the wells. Quantification of the optical density was performed using a spectrophotometer (Infinite M Nano Tecan group ldt.; Mannedorf, Switzerland) on a wavelength of 450 nm. For the stool antigen ELISA test, a cutoff of 3 ng/mL was used as per the manufacturers’ protocol.

For measurement of the FIT (OC-Sensor, Eiken) samples, the same procedure was followed, except for sample preparation. For FIT, 100uL of undiluted, centrifuged FIT fluid was used. Clean FIT fluid was compared to the assay buffer to confirm there was no interference of the FIT assay buffer on the procedure (not shown).

### Sample size

Based on previous reports, we estimated the prevalence of *H. pylori* in the Dutch population at 30% [16]. Sensitivity rates for the different non-invasive tests for *H. pylori* are 90–96% for the UBT, 86–94% for serological testing, and 81–98% for SAT. The sensitivity of Hp-FIT was unknown. Similar sensitivity rate as for the SAT was used (92%) to perform power calculations using the UBT as the reference standard. For a one-sided non-inferiority margin of 10%, a total of 55 *H. pylori-*positive subjects and 110 controls were required (using a ratio of 1:2) to have 80% power to detect an effect for which the upper limit of a one-sided 95% confidence interval will exclude a difference in favor of the standard test of more than 10%.

### Statistical analyses

Positivity rate (PR) was defined as the proportion of positive tests in participants with an analyzable test. The positive predictive value (PPV) comprised all participants diagnosed with *H. pylori* UBT by the studied test proportionally to participants with a positive *H. pylori* UBT result. The negative predictive value (NPV) comprised all participants with a negative *H. pylori* UBT result by the studied test proportionally to participants with a negative *H. pylori* UBT result. Sensitivity was calculated by dividing true positives by true positives plus false negative results, multiplied by 100. Specificity was calculated by dividing true negatives by true negatives plus false positives, multiplied by 100. Overall accuracy was calculated by dividing true positives and true negatives by all performed tests. Confidence intervals for sensitivity, specificity and accuracy are “exact” Clopper–Pearson confidence intervals. For all tests, receiver operating characteristic (ROC) curves with their area under the curve (AUC) were calculated. An AUC > 0.9 was considered as “outstanding discrimination”, 0.8–0.9 as “excellent discrimination”, 0.7–0.8 as “acceptable discrimination”, 0.5–0.7 as “poor discrimination” and 0.5 as “no discrimination” [17]. Differences between categorical variables, such as patient preferences in questionnaires, were evaluated using a Chi-squared test or McNemar test when appropriate. Differences between means were evaluated using a *t* test. A two-sided significance level of *p* < 0.05 for all tests was used.

## Results

### Baseline characteristics

**Fig. 1:**
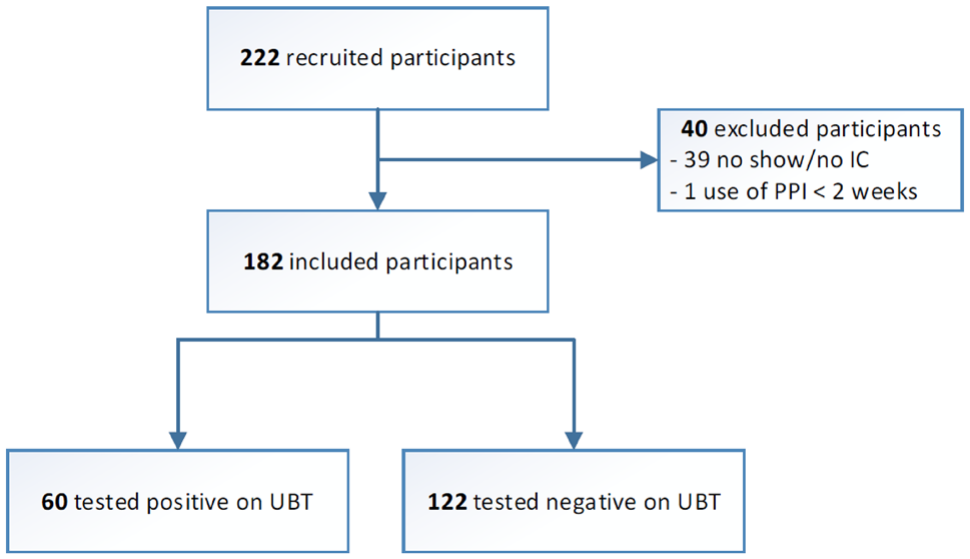
Flowchart of study inclusions; *PPI* proton pump inhibitor, *IC*; informed consent, *UBT* urea breath test

In total, 222 patients were considered for this study of which 182 patients were included based on the inclusion and exclusion criteria (37.4% male, median age 52.4 years (IQR 22.4)) (Fig. 1). Of these, 60 (33.0%) tested positive for *H. pylori* by UBT (Table 1).

**Table 1:** Baseline characteristics of the study population

	Study participants (*n* = 182)
Sex: male (%)	68 (37.4)
Age: median (IQR)	52.4 (22.4)
*Ethnicity n (%)*	
Western Europe	110 (61.0)
Middle East	12 (6.5)
Western Africa	9 (4.8)
Latin America	7 (3.7)
Asian	8 (4.3)
Missing	36 (19.7)
*Indication of UBT n (%)*	
Diagnostic	53 (35.8)
Eradication	76 (51.4)
Lynch Screening	19 (12.8)
Positive test *n* (%)	60 (33.0)
*Complete sampling n (%)*	
FIT	161 (88.5)
SAT	149 (81.8)
Serum	159 (87.4)

*FIT* fecal immunochemical test, *IQR* inter quartile range, *UBT* urea breath test, *SAT* stool antigen test

### Test accuracy

**Fig. 2:**
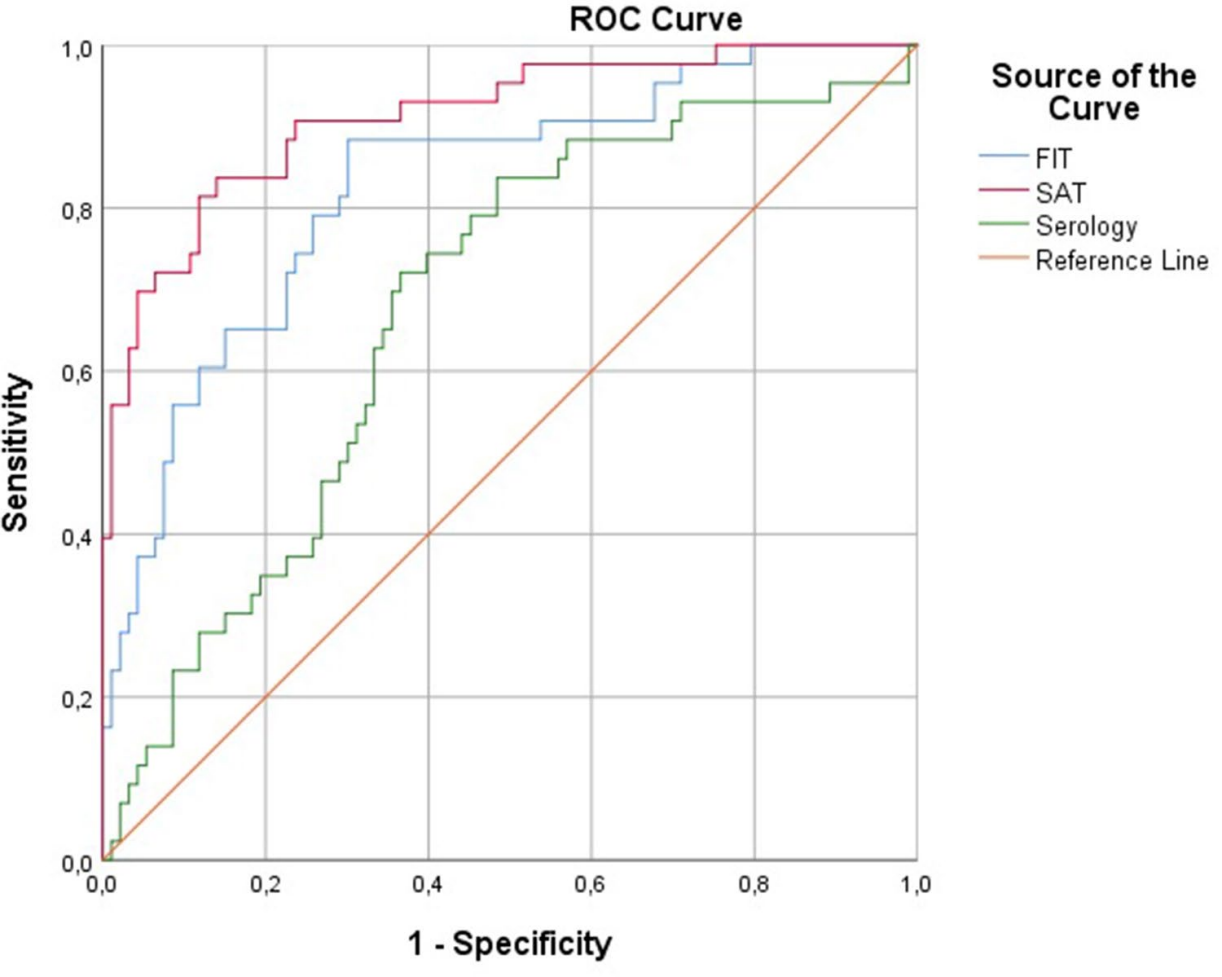
ROC curves for all tests *AUC *area under the curve, *FIT* fecal immunochemical test, *ROC* receiver operating characteristics, *SAT* stool antigen test

All tests were plotted in a ROC curve (Fig. 2). The SAT showed the highest AUC with 0.91, and the Hp-FIT showed an AUC of 0.85. The serology test had an AUC of 0.68.

### Cutoff points

Since the use of FIT in this context is newly investigated, a cutoff is not yet established. Table 2 shows all test outcomes for Hp-FIT when the same cutoff is used as for SAT (i.e., 3 ng/mL). Under these conditions, SAT and Hp-FIT showed comparable overall accuracy: 71.1% (95%CI 63.2–78.3) vs. 77.6% (95%CI 70.4–83.8), respectively (*p* = 0.97). The sensitivity rate for SAT was 91.8% (95%CI 80.4–97.7) versus 94.2% (95%CI 84.1–98.9) for Hp-FIT (*p* = 0.998). Both tests however had a low specificity rate of 61.0% (95%CI 50.7–70.6) and 69.7% (95%CI 60.2–78.1) for SAT and Hp-FIT, respectively (*p* = 0.442). The serology test scored low on all primary outcomes, with an overall accuracy rate of 49.7% (95%CI 41.7–57.7).

**Table 2:** Primary outcomes measures of all tests at a cutoff of 3 ng/mL for SAT and Hp-FIT and 30 EIU for serology

Test	PR (%) (95%CI)	PPV (%) (95%CI)	NPV (%) (95%CI)	Sensitivity (%) (95%CI)	Specificity (%) (95%CI)	Accuracy (%) (95%CI)
Hp-FIT	50.9 (43.0–58.9)	59.8 (52.6–66.6)	96.2 (89.4–98.7)	94.2 (84.1–98.9)	69.7 (60.2–78.1)	77.6 (70.4–83.8)
SAT	56.4 (48.0–64.5)	53.6 (47.1–59.9)	93.9 (85.5–97.5)	91.8 (80.4–97.7)	61.0 (50.7–70.6)	71.1 (63.2–78.3)
Serology	73.6 (66.0–80.3)	35.9 (32.3–39.7)	88.1 (75.6–94.6)	89.4 (76.9–96.5)	33.0 (24.4–42.6)	49.7 (41.7–57.7)

*CI* confidence interval, *FIT* fecal immunochemical test, *NPV* negative predictive value, *PPV* positive predictive value, *PR* positivity rate, *SAT* stool antigen test

The means of absolute stool antigen concentration were compared for Hp-FIT and SAT for false positive and true positive test results. Absolute stool *H. pylori* antigen concentration in false positive Hp-FIT versus true positive Hp-FIT was 9.3 ng/mL (95%CI 8.6–12.3) vs 30.9 ng/mL (95%CI 19.0–45.3) (*p* < 0.001), respectively. For SAT, this was 8.6 ng/mL (95%CI 5.4–9.8) for false positives and 46.2 ng/mL (95%CI 32.4–58.3) for true positives (*p* < 0.001).

Choosing different cutoff levels affects performance of the test. Test outcomes at different cutoff points for Hp-FIT are therefore shown in Table 3. When the cutoff of Hp-FIT was raised up to 4 ng/mL or higher, the overall accuracy was lower. By raising the cutoff up to 6 ng/mL specificity rate increased to 74.6% (95%CI 65.9–82.0); however, this came at the cost of a considerable decrease in the sensitivity rate (67.3% 95%CI 53.3–79.3). Lowering the cutoff to 2 ng/mL resulted in a lower overall accuracy due to a decrease in specificity rate (47.5% 95%CI 38.4–56.8).

**Table 3:** Test outcomes of FIT with different cutoffs*,*

Cutoff (ng/mL)	PR (%) (95%CI)	PPV (%) (95%CI)	NPV (%) (95%CI)	Sensitivity (%) (95%CI)	Specificity (%) (95%CI)	Accuracy (%) (95%CI)
2	62.1 (54.1–69.6)	47.1 (42.7–51.6)	95.1 (86.3–98.3)	95.0 (86.1–99.0)	47.5 (38.4–56.8)	63.2 (55.7–70.2)
4	44.7 (36.9–52.8)	57.0 (50.3–63.5)	92.1 (85.2–96.0)	88.3 (77.4–95.2)	67.2 (58.1–75.4)	74.2 (67.2–80.4)
5	39.1 (31.6–47.1)	59.5 (52.0–66.7)	89.8 (83.2–94.0)	83.3 (71.5–91.7)	72.1 (63.3–79.9)	75.8 (68.9–81.9)
6	34.2 (26.9–42.0)	59.2 (50.9–67.0)	85.9 (79.5–90.5)	67.3 (53.3–79.3)	74.6 (65.9–82.0)	74.7 (67.8–80.9)

*CI* confidence interval, *FIT* fecal immunochemical test, *NPV* negative predictive value, *PPV* positive predictive value, *PR* positivity rate

### Patient preferences

**Fig. 3:**
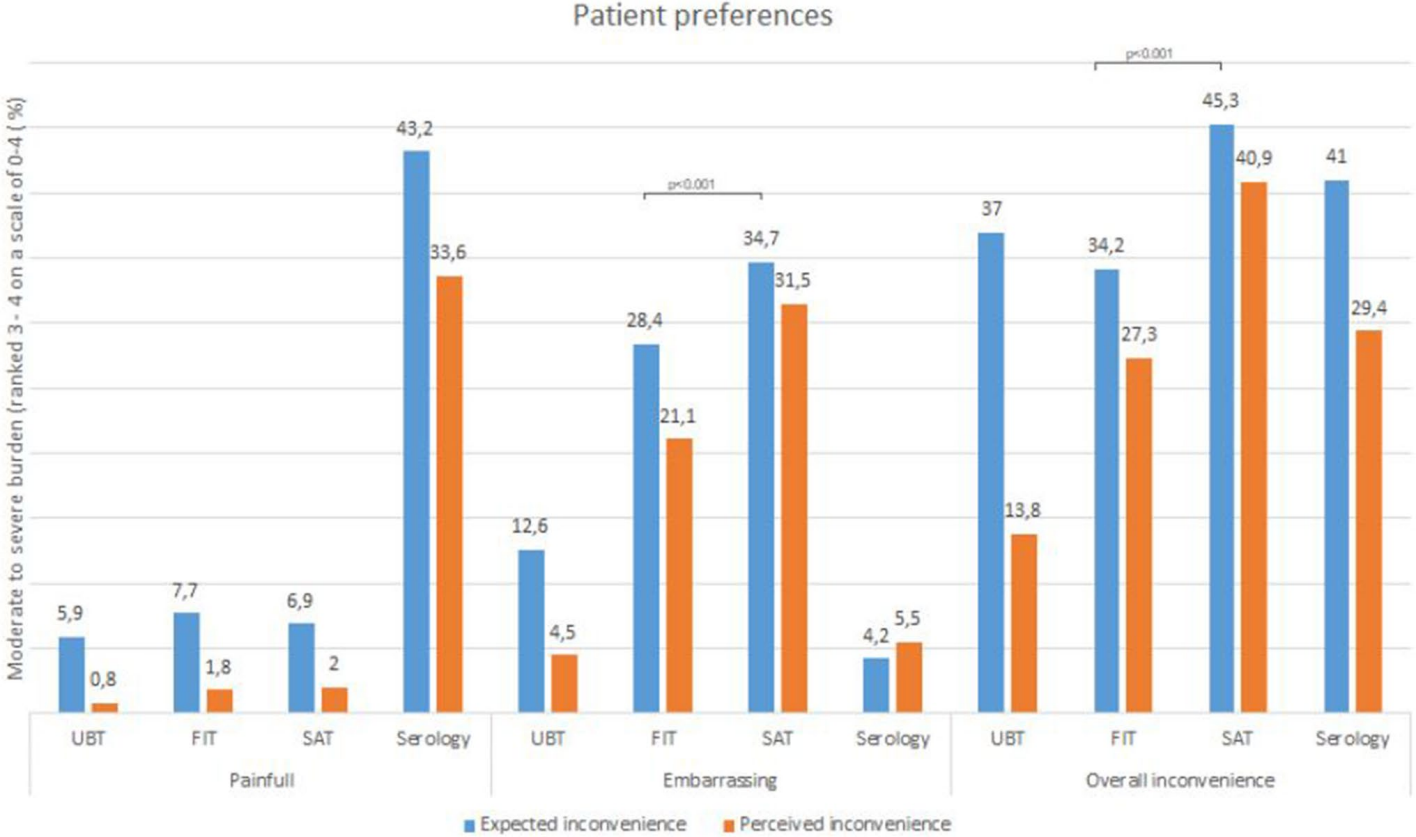
Histogram of patient preferences retrieved through questionnaires *UBT* urea breath test, *Hp-FIT* fecal immunochemical test for *H. pylori*

Expected and perceived burdens were compared for all participants using questionnaires before and after performing all tests. UBT was rated best, with only 13.8% of the participants perceiving moderate to severe overall inconvenience (ranking 3 or 4 on a scale from 0 to 4 with 0 being “no burden” and 4 “ severe burden”), followed by Hp-FIT with 27.3%, the serum test 29.4% and lastly SAT with 40.9% (Fig. 3). Inconvenience in SAT was mostly due to embarrassment due to the execution of the test (scooping feces (SAT) vs. picking feces (Hp-FIT)). Expected and perceived convenience was similar across most aspects. Overall, UBT was perceived as more convenient than expected.

## Discussion

We investigated FIT as a new non-invasive test for diagnosis of *H pylori* infection and compared the outcome of Hp*-*FIT directly to the other available non-invasive diagnostic *H. pylori* tests. This is the first study to show that it is possible to determine *H. pylori* antigen in FIT. Furthermore, we show that Hp*-*FIT has comparable accuracy and sensitivity rates to SAT and was perceived as a more convenient test. This study is an important stepping stone toward (cost-efficient) combined upper and lower gastrointestinal pre-cancerous screening. As the FIT test is already widely used in current practice, it can therefore easily be adopted for such an expanded indication in general practitioners’ offices, hospitals as well as in screening programs.

In previous studies, diagnostic tests for diagnosis of *H. pylori* showed that accuracy rates of stool antigen tests using ELISA are comparable with UBT [8, 9]. The current Maastricht V consensus report therefore states that SAT and UBT can be used interchangeably [2]. Our results showed lower specificity rates. This might be due to the fact that 45% of the study population underwent eradication therapy prior to testing. It is known that this could affect the accuracy of stool antigen tests [9].

Both Hp-FIT and SAT showed a high rate of false positivity. This has been established before in a Cochrane meta-analysis in which the results of 101 different studies were compared [18]. Unfortunately, all but one of these studies were of poor methodological quality, for which reason only a suboptimal indirect comparison could be made. A few possible explanations for the high rate of false positives were discussed in this meta-analysis. First, UBT was considered the golden standard for *H. pylori* positivity. However even though UBT is an outstanding test, false negative UBT results do occur [19]. Second, there was large heterogeneity of cutoff points used for each of the tests. The current study investigated the accuracy rates at different cutoff points. The currently used cutoff point of 3 ng/mL showed the most favorable accuracy rates. However, different cutoff points might be preferred for different purposes such as *H. pylori* eradication verification tests compared to diagnostic tests or screening purposes. For actual implementation of Hp-FIT, screening issues such as subsequent intervention after a positive test should be addressed (i.e., esophagogastroduodenoscopy, direct eradication therapy) taking into account already available guidelines per country.

As already stated, for a test to be widely adopted and effective, ease of use and non-invasiveness are likely as important as test accuracy [10]. Therefore, this study also investigated patient preferences. The UBT showed to have the best overall convenience. When both fecal tests were compared, Hp-FIT appeared to be perceived as most convenient. From previous CRC screening studies, we already have learned that feces tests with a pricker instead of a scoop are more convenient to use [11]. There are some clear benefits of using SAT or Hp-FIT over UBT in particular for patients, since the test can easily be performed at home, and also particularly from a socioeconomic point of view, since there is no need for advanced expensive technical materials or direct contact with a technician or nurse.

This study has several limitations. First, the UBT was used as the golden standard instead of biopsy confirmation. This might skew the overall results by a small margin of a 96% sensitivity of UBT compared to biopsy testing [2]. This happens most profoundly in the case of concurrent use of PPIs [20]. Recent use of PPIs or bismuth was therefore an exclusion criterion in our study. Furthermore, UBT is known to produce rare false positive results in the presence of non-*H. pylori* urease-producing bacteria or fungi ( i.e., *Proteus mirabilis, Citrobacter freundii, Klebsiella pneumoniae, Enterobacter cloacae* and *Staphylococcus aureus*) in either the stomach or the oral cavity [21, 22.

Second, for Hp-FIT analysis 100uL of undiluted, centrifuged FIT fluid was used. This might not always fully correspond with the same amount of feces. Hence, this also might affect test results. However, our study end points are based on binomial results (either positive or negative for *H. pylori*), which makes the absolute amount of feces per test of less relevance. Future studies should compare different kits for both FIT and SAT. Third, patient preferences results might be biased since study dropouts could not be questioned about preferences. Fourth, the use of PPI, antibiotics or bismuth was an exclusion criterion of the study tested through a questionnaire. This might cause reporting bias. In a real life (screening) setting, the use of PPIs will not always be ceased and therefore will affect accuracy.

This study is the first to show that Hp-FIT can be used as a new and convenient test in daily practice. It is an important step in screening, being the first step toward a potential cost-efficient, dual screening program of the upper and lower gastrointestinal tract.

## Supplementary Information

Below is the link to the electronic supplementary material.

Supplementary file1 (DOC 563 KB)

## Abbreviations

CRC: Colorectal cancer
ELISA: Enzyme immune assay
FIT: Fecal immunochemical test
*H. pylori*: *Helicobacter pylori*
PPI: Proton pump inhibitor
ROC: Receiving operating curve
UBT: Urea breath test
SAT: Stool antigen test
WHO: World Health Organization
