# Supplementary material for: Accuracy of H. pylori fecal antigen test using fecal immunochemical test (FIT)
Source: Gastric Cancer. 2021 Nov 18;25(2):375–81. doi: 10.1007/s10120-021-01264-8 (PMC8882108; doi:10.1007/s10120-021-01264-8)
Supplement: Supplementary file 1 — Supplementary file1 (DOC 563 KB) [file 10120_2021_1264_MOESM1_ESM.doc]

**S1 Questionnaires (in Dutch)**


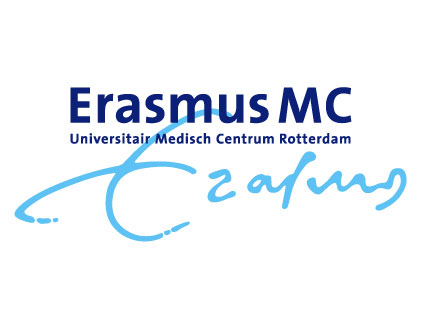


**VRAGENLIJST**

**Helicobacter pylori bepaling in FIT**

**- Ervaringen -**

**Toelichting op deze vragenlijst**

U heeft onlangs verschillende onderzoeken ondergaan voor een Helicobacter Pylori bepaling. Graag willen wij weten hoe u dit heeft ervaren. Wij willen u daarom vragen bijgevoegde vragenlijst in te vullen. Het is voor ons erg belangrijk om te weten hoe u over de verschillende onderzoeken denkt, zodat wij in de toekomst goede informatie kunnen geven over deze onderzoeken. Verder hopen we hiermee de kwaliteit van het onderzoek te kunnen verbeteren.

Het kan soms lijken alsof een vraag voor u niet van toepassing of overbodig is, toch verzoeken we u alle vragen te beantwoorden. Mocht u twijfelen, geef dan het antwoord dat het dichtst in de buurt van uw persoonlijke situatie komt. Uw vragenlijst zal anoniem worden verwerkt.

**Invulinstructie**

• In deze vragenlijst kunt u uw antwoord geven door een antwoordvakje *aan te kruisen*.

• Als u al een vakje heeft aangekruist en u wilt uw antwoord wijzigen, dan moet het foutief aangekruiste vakje geheel zwart/blauw gemaakt worden – vervolgens kunt u het juiste antwoordvakje aankruisen.

• Bij vragen waar u zelf iets moet opschrijven, schrijft u dan *in blokletters*.

• Wij verzoeken u alle vragen te beantwoorden.

Met *reguliere ontlastingstest (SAT)* wordt bedoeld:


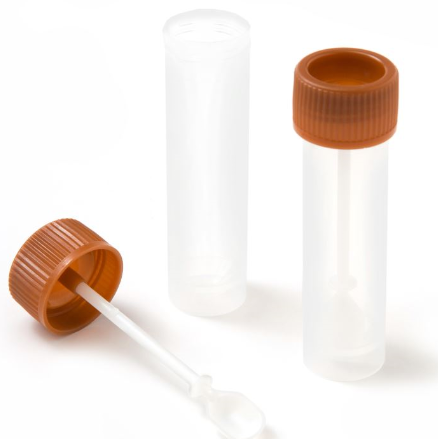


Met *de te onderzoeken test (FIT)* wordt bedoeld:

[
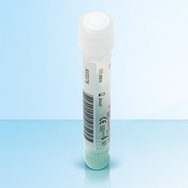
](http://www.google.nl/url?sa=i&rct=j&q=&esrc=s&source=images&cd=&cad=rja&uact=8&ved=0ahUKEwiwn4n3x8nVAhXBZlAKHcETAlsQjRwIBw&url=http%3A%2F%2Fwww.sysmex-europe.com%2Fproducts%2Foncology%2Ffaecal-immunological-test-for-haemaoglobin%2Ffit-in-symptomatic-testing.html&psig=AFQjCNFCikH5JnQ2t_uyMvLZT6dsvSgjzg&ust=1502347824010937)

**Hartelijk dank voor uw medewerking.**

Mw. Dr. M.C.W. Spaander, Maag-, Darm-, Leverarts, Erasmus MC

Drs. S.A.V. Nieuwenburg, arts-onderzoeker Maag- Darm- en Leverziekten, Erasmus MC

**Voor vragen over deze vragenlijst of over eventuele deelname aan het onderzoek kunt u bellen met de studietelefoon, te bereiken op werkdagen op telefoonnummer 06-81415567**

| **Vragenlijst Hp bepaling in FIT** |
| --- |

| Onderstaande vragen gaan over uw verwachtingen van de reeds ondergane onderzoeken voor de Helicobacter pylori bepaling in bloed, ontlasting en uitgeademde lucht. Wilt u bij elke uitspraak aangeven in hoeverre u het eens dan wel oneens bent met de volgende uitspraken. Het is belangrijk dat u hierbij uw eigen mening of gevoel weergeeft. Er zijn geen goede of foute antwoorden. | | | | | | | | |
| --- | --- | --- | --- | --- | --- | --- | --- | --- |
| 1. | **In hoeverre vond u de ademtest belastend?** | | | | | | | |
|  | **□** | **□** | **□** | **□** | | **□** | | |
|  | helemaal niet belastend | een beetje belastend | Enigszins belastend | Tamelijk belastend | | Zeer  belastend | | |
| 2. | **In hoeverre vond u de ademtest pijnlijk?** | | | | | | | |
|  | **□** | **□** | **□** | | **□** | **□** | | |
|  | Helemaal niet pijnlijk | Een beetje pijnlijk | Enigszins pijnlijk | | Tamelijk pijnlijk | Zeer pijnlijk | | |
| 3. | **In hoeverre vond u de ademtest beschamend/gênant?** | | | | | | | |
|  | **□** | **□** | **□** | **□** | | | **□** | |
|  | helemaal niet beschamend | een beetje beschamend | enigszins beschamend | tamelijk beschamend | | | | zeer beschamend |

| 4. | **In hoeverre vond u de reguliere ontlastingstest (SAT) belastend?** | | | | | | | |
| --- | --- | --- | --- | --- | --- | --- | --- | --- |
|  | **□** | **□** | **□** | **□** | | **□** | | |
|  | helemaal niet belastend | een beetje belastend | Enigszins belastend | Tamelijk belastend | | Zeer  belastend | | |
| 5. | **In hoeverre vond u de reguliere ontlastingstest (SAT) pijnlijk?** | | | | | | | |
|  | **□** | **□** | **□** | | **□** | **□** | | |
|  | Helemaal niet pijnlijk | Een beetje pijnlijk | Enigszins pijnlijk | | Tamelijk pijnlijk | Zeer pijnlijk | | |
| 6. | **In hoeverre vond u de reguliere ontlastingstest (SAT) beschamend/gênant?** | | | | | | | |
|  | **□** | **□** | **□** | **□** | | | **□** | |
|  | helemaal niet beschamend | een beetje beschamend | enigszins beschamend | tamelijk beschamend | | | | zeer beschamend |
| 7. | **In hoeverre vond u de te onderzoeken test (FIT)** **belastend?** | | | | | | | |
|  | **□** | **□** | **□** | **□** | | **□** | | |
|  | helemaal niet belastend | een beetje belastend | Enigszins belastend | Tamelijk belastend | | Zeer  belastend | | |
| 8. | **In hoeverre vond u de te onderzoeken test (FIT)** **pijnlijk?** | | | | | | | |
|  | **□** | **□** | **□** | | **□** | **□** | | |
|  | Helemaal niet pijnlijk | Een beetje pijnlijk | Enigszins pijnlijk | | Tamelijk pijnlijk | Zeer pijnlijk | | |
| 9. | **In hoeverre vond u de te onderzoeken test (FIT)** **beschamend/gênant?** | | | | | | | |
|  | **□** | **□** | **□** | **□** | | | **□** | |
|  | helemaal niet beschamend | een beetje beschamend | enigszins beschamend | tamelijk beschamend | | | | zeer beschamend |

| 10. | **In hoeverre vond u de bloedtest** **belastend?** | | | | | | | |
| --- | --- | --- | --- | --- | --- | --- | --- | --- |
|  | **□** | **□** | **□** | **□** | | **□** | | |
|  | helemaal niet belastend | een beetje belastend | Enigszins belastend | Tamelijk belastend | | Zeer  belastend | | |
| 11. | **In hoeverre vond u de bloedtest pijnlijk?** | | | | | | | |
|  | **□** | **□** | **□** | | **□** | **□** | | |
|  | Helemaal niet pijnlijk | Een beetje pijnlijk | Enigszins pijnlijk | | Tamelijk pijnlijk | Zeer pijnlijk | | |
| 12. | **In hoeverre vond u de bloedtest** **beschamend/gênant?** | | | | | | | |
|  | **□** | **□** | **□** | **□** | | | **□** | |
|  | helemaal niet beschamend | een beetje beschamend | enigszins beschamend | tamelijk beschamend | | | | zeer beschamend |

| Tot slot. | |
| --- | --- |
| 13. | **Wat is de datum van invullen van deze vragenlijst?** |
|  | **_____ - _____ - __________ (dag-maand-jaar)** |

**Wilt u alstublieft controleren of u alle vragen heeft ingevuld en geen bladzijden heeft overgeslagen.**

**HARTELIJK DANK VOOR UW MEDEWERKING**


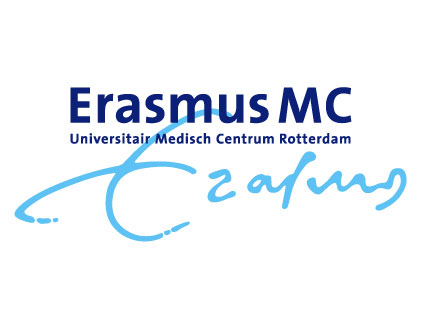


**VRAGENLIJST**

**Helicobacter pylori bepaling in FIT**

**- verwachtingen -**

**Toelichting op deze vragenlijst**

U heeft een uitnodiging ontvangen om mee te doen aan de studie die onderzoekt of de maagbacterie Helicobacter Pylori (Hp) in de FIT ontlastingstest bepaald kan worden. Graag willen wij weten wat u van onderzoek vindt.

Wij vinden uw mening erg belangrijk, ook als u besloten heeft om niet mee te doen of als u nog geen keuze gemaakt heeft.

Het is belangrijk dat de vragenlijst wordt ingevuld door degene aan wie de brief is geadresseerd. Controleer daarom of uw naam in de brief is vermeld. Is dit niet het geval, wilt u dan de vragenlijst aan de persoon geven voor wie deze is bestemd?

Er zijn geen goede of foute antwoorden, het gaat om uw persoonlijke ervaring. Uw vragenlijst zal anoniem worden verwerkt.

**Invulinstructie**

• In deze vragenlijst kunt u uw antwoord geven door een antwoordvakje *aan te kruisen*.

• Als u al een vakje heeft aangekruist en u wilt uw antwoord wijzigen, dan moet het

foutief aangekruiste vakje geheel zwart/blauw gemaakt worden – vervolgens kunt u het juiste antwoordvakje aankruisen.

• Bij vragen waar u zelf iets moet opschrijven, schrijft u dan *in blokletters*.

• Wij verzoeken u alle vragen te beantwoorden.

Met *reguliere ontlastingstest (SAT)* wordt bedoeld:


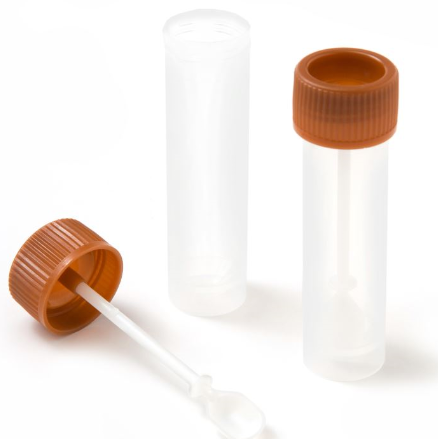


Met *de te onderzoeken test (FIT)* wordt bedoeld:

[
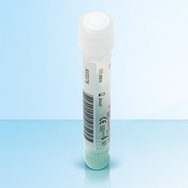
](http://www.google.nl/url?sa=i&rct=j&q=&esrc=s&source=images&cd=&cad=rja&uact=8&ved=0ahUKEwiwn4n3x8nVAhXBZlAKHcETAlsQjRwIBw&url=http%3A%2F%2Fwww.sysmex-europe.com%2Fproducts%2Foncology%2Ffaecal-immunological-test-for-haemaoglobin%2Ffit-in-symptomatic-testing.html&psig=AFQjCNFCikH5JnQ2t_uyMvLZT6dsvSgjzg&ust=1502347824010937)

**Hartelijk dank voor uw medewerking.**

Mw. Dr. M.C.W. Spaander, Maag-, Darm-, Leverarts, Erasmus MC

Mw. Drs. S.A.V. Nieuwenburg, arts-onderzoeker Maag- Darm- en Leverziekten, Erasmus MC

**Voor vragen over deze vragenlijst of over eventuele deelname aan het onderzoek kunt u bellen met de studietelefoon, te bereiken op werkdagen op telefoonnummer 06-50033983**

| **Vragenlijst Hp bepaling in FIT** | | | | | |
| --- | --- | --- | --- | --- | --- |
|  |  | | | | |
| 1. | **Hoe duidelijk vond u de uitleg over het onderzoek?** | | | | |
|  | **□** | **□** | **□** | **□** | **□** |
|  | zeer duidelijk | duidelijk | onduidelijk | zeer onduidelijk | niet gelezen |
| 2. | **Waarover had u graag meer informatie gekregen? (*U mag meerdere antwoorden geven*)** | | | | |
|  | **□** | het doel van het onderzoek | | | |
|  | **□** | hoe vaak maagaandoeningen voorkomen | | | |
|  | **□** | de voordelen van deelname | | | |
|  | **□** | de nadelen van deelname | | | |
|  | **□** | de vrijwilligheid van deelname | | | |
|  | **□** | niets, de informatie was voor mij voldoende | | | |
|  | **□** | anders, namelijk… | | | |

| Onderstaande vragen gaan over uw verwachtingen van de te ondergane onderzoeken voor de Helicobacter pylori bepaling in bloed, ontlasting en uitgeademde lucht. Wilt u bij elke uitspraak aangeven in hoeverre u het eens dan wel oneens bent met de volgende uitspraken. Het is belangrijk dat u hierbij uw eigen mening of gevoel weergeeft. Er zijn geen goede of foute antwoorden. | | | | | | | | |  |
| --- | --- | --- | --- | --- | --- | --- | --- | --- | --- |
| 1. | **In hoeverre verwacht u dat de ademtest belastend zal zijn?** | | | | | | | | |
|  | **□** | **□** | **□** | **□** | | **□** | | | |
|  | helemaal niet belastend | een beetje belastend | Enigszins belastend | Tamelijk belastend | | Zeer  belastend | | | |
| 2. | **In hoeverre verwacht u dat de ademtest pijnlijk zal zijn?** | | | | | | | | |
|  | **□** | **□** | **□** | | **□** | **□** | | | |
|  | Helemaal niet pijnlijk | Een beetje pijnlijk | Enigszins pijnlijk | | Tamelijk pijnlijk | Zeer pijnlijk | | | |
| 3. | **In hoeverre verwacht u dat de ademtest beschamend/gênant zal zijn?** | | | | | | | | |
|  | **□** | **□** | **□** | **□** | | | **□** | | |
|  | helemaal niet beschamend | een beetje beschamend | enigszins beschamend | tamelijk beschamend | | | | zeer beschamend | |

| 4. | **In hoeverre verwacht u dat de reguliere ontlastingstest (SAT) belastend zal zijn?** | | | | | |
| --- | --- | --- | --- | --- | --- | --- |
|  | **□** | **□** | **□** | **□** | | **□** |
|  | helemaal niet belastend | een beetje belastend | Enigszins belastend | Tamelijk belastend | | Zeer  belastend |
| 5. | **In hoeverre verwacht u dat de reguliere ontlastingstest (SAT) pijnlijk zal zijn?** | | | | | |
|  | **□** | **□** | **□** | | **□** | **□** |
|  | Helemaal niet pijnlijk | Een beetje pijnlijk | Enigszins pijnlijk | | Tamelijk pijnlijk | Zeer pijnlijk |

| 6. | **In hoeverre verwacht u de reguliere ontlastingstest (SAT) beschamend/gênant zal zijn?** | | | | | | | |
| --- | --- | --- | --- | --- | --- | --- | --- | --- |
|  | **□** | **□** | **□** | **□** | | | **□** | |
|  | helemaal niet beschamend | een beetje beschamend | enigszins beschamend | tamelijk beschamend | | | | zeer beschamend |
| 7. | **In hoeverre verwacht u dat de te onderzoeken test (FIT)** **belastend zal zijn?** | | | | | | | |
|  | **□** | **□** | **□** | **□** | | **□** | | |
|  | helemaal niet belastend | een beetje belastend | Enigszins belastend | Tamelijk belastend | | Zeer  belastend | | |
| 8. | **In hoeverre verwacht u dat de te onderzoeken test (FIT)** **pijnlijk zal zijn?** | | | | | | | |
|  | **□** | **□** | **□** | | **□** | **□** | | |
|  | Helemaal niet pijnlijk | Een beetje pijnlijk | Enigszins pijnlijk | | Tamelijk pijnlijk | Zeer pijnlijk | | |
| 9. | **In hoeverre verwacht u de te onderzoeken test (FIT)** **beschamend/gênant zal zijn?** | | | | | | | |
|  | **□** | **□** | **□** | **□** | | | **□** | |
|  | helemaal niet beschamend | een beetje beschamend | enigszins beschamend | tamelijk beschamend | | | | zeer beschamend |
| 10. | **In hoeverre verwacht u dat de bloedtest belastend zal zijn?** | | | | | | | |
|  | **□** | **□** | **□** | **□** | | **□** | | |
|  | helemaal niet belastend | een beetje belastend | Enigszins belastend | Tamelijk belastend | | Zeer  belastend | | |
| 11. | **In hoeverre verwacht u dat de bloedtest pijnlijk zal zijn?** | | | | | | | |
|  | **□** | **□** | **□** | | **□** | **□** | | |
|  | Helemaal niet pijnlijk | Een beetje pijnlijk | Enigszins pijnlijk | | Tamelijk pijnlijk | Zeer pijnlijk | | |
| 12. | **In hoeverre verwacht u dat de bloedtest beschamend/gênant zal zijn?** | | | | | | | |
|  | **□** | **□** | **□** | **□** | | | **□** | |
|  | helemaal niet beschamend | een beetje beschamend | enigszins beschamend | tamelijk beschamend | | | | zeer beschamend |

| Tot slot nog enkele vragen over uzelf. | | |
| --- | --- | --- |
| 13. | **Wat is uw burgerlijke staat?** | |
|  | **□** | alleenstaand |
|  | **□** | samenwonend/gehuwd |
|  | **□** | duurzame relatie, maar niet samenwonend |
|  | **□** | anders, namelijk… |
| 14. | **Welke situatie is voor u op dit moment het meest van toepassing?** | |
|  | **□** | ik heb betaald werk voor ____ uur per week (*graag invullen*) |
|  | **□** | ik doe geen betaald werk want ik zorg voor de huishouding en evt. kinderen |
|  | **□** | ik doe geen betaald werk vanwege gezondheidsproblemen |
|  | **□** | ik doe geen betaald werk om andere redenen (bijv. onvrijwillig werkloos, vrijwilligerswerk) |
|  | **□** | ik ben gepensioneerd of met de VUT |
| 15. | **Tot welke bevolkingsgroep voelt u zich behoren?** | |
|  | **□** | Nederlands |
|  | **□** | Turks |
|  | **□** | Marokkaans |
|  | **□** | Hindoestaans |
|  | **□** | Creools |
|  | **□** | Surinaams |
|  | **□** | anders, namelijk… |

| 16. | | **Welke taal spreekt u thuis?** | | | | | | |
| --- | --- | --- | --- | --- | --- | --- | --- | --- |
|  | | **□** | alleen Nederlands | | | | | |
|  | | **□** | Nederlands en een andere taal | | | | | |
|  | | **□** | andere taal, namelijk… | | | | | |
| 17. | | **Heeft u moeite bij het lezen van kranten, brieven of folders in het Nederlands?** | | | | | | |
|  | | **□** | ja, altijd | | | | | |
|  | | **□** | ja, vaak | | | | | |
|  | | **□** | ja, soms | | | | | |
|  | | **□** | nee, nooit | | | | | |
| 18. | | **Kent u mensen die (ook) hebben meegedaan aan dit onderzoek** | | | | | | |
|  | | **□** | | | | **□** | | |
|  | | ja | | | | nee | | |
| 19. | | **Hoe vaak bezoekt u gemiddeld een dokter? *(uitgezonderd de artsen die u in het kader van dit onderzoek ziet)*** | | | | | | |
|  | | **□** | ongeveer 1 maal per maand of vaker | | | | | |
|  | | **□** | om de paar maanden | | | | | |
|  | | **□** | ongeveer 1 maal per jaar | | | | | |
|  | | **□** | eens in de 2 tot 5 jaar | | | | | |
| 20. | **Hoe zou u over het algemeen uw gezondheid noemen?** | | | | | | | |
|  | **□** | | | **□** | **□** | | **□** | **□** |
|  | slecht | | | matig | goed | | zeer goed | Uitstekend |
|  |  | | |  |  | |  |  |
| 21. | | **Heeft u wel eens last van:** | | | | | | |
|  | | Zuurbranden **□**  ja | | | | **□**  nee | | |
|  | | Misselijkheid **□** | | | | **□** | | |
|  | | ja | | | | nee | | |
|  | | Braken **□**  ja | | | | **□**  nee | | |
|  | | Snel vol gevoel **□**  ja | | | | **□**  nee | | |
|  | | Pijn in de maag **□**  ja | | | | **□** nee | | |
|  | | Andere klachten **□**  Ja, namelijk: | | | | **□**  nee | | |
| 22. | | **Gebruikt u pijnstillers zoals ibuprofen/diclofenac/neurofen/advil/aspirine? (NSAID’s)** | | | | | | |
|  | | **□** | | | | **□** | | |
|  | | Ja, namelijk .. keer/week | | | | nee | | |
| 23. | | **Gebruikt u zuurremmers zoals Rennies, gaviscon, omeprazol, pantoprazol, nexium, pariet?** | | | | | | |
|  | | **□** | | | | **□** | | |
|  | | Ja, namelijk .. keer/week | | | | Nee | | |
| 24. | | **Bent u ooit eerder behandeld met antibiotica vanwege een maagbacterie?** | | | | | | |
|  | | **□** | | | | **□** | | |
|  | | Ja, geschatte maand/jaartal: | | | | nee | | |
|  | |  | | | |  | | |
| 25. | | **Rookt u?** | | | | | | |
|  | | **□** | | | | **□ □** | | |
|  | | Ja, namelijk .. sig/per dag, sinds 19.. | | | | nee niet meer, gestopt sinds 19.. | | |

| 26. | **Drinkt u alcohol?** | |
| --- | --- | --- |
|  | **□** | **□** |
|  | Ja, namelijk .. glazen/week | Nee |

| 27. | **Komen er in de familie maagklachten voor?** | |
| --- | --- | --- |
|  | **□** | **□** |
|  | Ja, namelijk bij.. | nee |
| 28. | **Is er bij een familielid wel eens een antibioticakuur voorgeschreven voor een maagbacterie?** | |
|  | **□** | **□** |
|  | Ja, namelijk bij .. | nee |
| 29. | **Komt er in de familie maagkanker voor?** | |
|  | **□** | **□** |
|  | Ja, namelijk bij .. | nee |

| Tot slot. | |
| --- | --- |
| 30. | **Wat is de datum van invullen van deze vragenlijst?** |
|  | **_____ - _____ - __________ (dag-maand-jaar)** |

**Wilt u alstublieft controleren of u alle vragen heeft ingevuld en geen bladzijden heeft overgeslagen.**

**HARTELIJK DANK VOOR UW MEDEWERKING**
